# Knowledge brokering between researchers and policymakers in Fiji to develop policies to reduce obesity: a process evaluation

**DOI:** 10.1186/1748-5908-8-74

**Published:** 2013-07-01

**Authors:** Gade Waqa, Helen Mavoa, Wendy Snowdon, Marj Moodie, Jimaima Schultz, Marita McCabe, Peter Kremer, Boyd Swinburn

**Affiliations:** 1 Pacific Research Centre for the Prevention of Obesity and Non-Communicable Diseases (C-POND), College of Medicine, Nursing and Health Sciences, Fiji National University, Tamavua Campus, Princes Road Tamavua, Suva, Fiji; 2 WHO Collaborating Centre for Obesity Prevention, Deakin University, 221 Burwood Highway, Burwood, 3125, Melbourne, Victoria, Australia; 3 Deakin Health Economics, Deakin University, 221 Burwood Highway, Burwood, 3125, Melbourne, Victoria, Australia; 4 National Food and Nutrition Centre, Ministry of Health, 1 Clarke Street, Suva, Fiji; 5 School of Psychology, Deakin University, Melbourne, Burwood Campus, 221 Burwood Highway, VIC 3125, Melbourne, Australia; 6 School of Exercise and Nutrition Sciences, Deakin University, 75 Pigdons Road, Waurn Ponds, Victoria, Australia; 7 School of Population Health, University of Auckland, 261 Morrin Rd, Auckland, Glen Innes, 1072, New Zealand

**Keywords:** Knowledge Exchange, Knowledge Brokering, Evidence-informed Policymaking, Evidence-informed Decisionmaking, Obesity Prevention

## Abstract

**Background:**

The importance of using research evidence in decisionmaking at the policy level has been increasingly recognized. However, knowledge brokering to engage researchers and policymakers in government and non-government organizations is challenging. This paper describes and evaluates the knowledge exchange processes employed by the Translational Research on Obesity Prevention in Communities (TROPIC) project that was conducted from July 2009 to April 2012 in Fiji. TROPIC aimed to enhance: the evidence-informed decisionmaking skills of policy developers; and awareness and utilization of local and other obesity-related evidence to develop policies that could potentially improve the nation’s food and physical activity environments. The specific research question was: Can a knowledge brokering approach advance evidence-informed policy development to improve eating and physical activity environments in Fiji.

**Methods:**

The intervention comprised: recruiting organizations and individuals; mapping policy environments; analyzing organizational capacity and support for evidence-informed policymaking (EIPM); developing EIPM skills; and facilitating development of evidence-informed policy briefs. Flexible timetabling of activities was essential to accommodate multiple competing priorities at both individual and organizational levels. Process diaries captured the duration, frequency and type of each interaction and/or activity between the knowledge brokering team and participants or their organizations.

**Results:**

Partnerships were formalized with high-level officers in each of the six participating organization. Participants (n = 49) developed EIPM skills (acquire, assess, adapt and apply evidence) through a series of four workshops and applied this knowledge to formulate briefs with ongoing one-to-one support from TROPIC team members. A total of 55% of participants completed the 12 to18 month intervention, and 63% produced one or more briefs (total = 20) that were presented to higher-level officers within their organizations. The knowledge brokering team spent an average of 30 hours per participant during the entire TROPIC process.

**Conclusions:**

Active engagement of participating organizations from the outset resulted in strong individual and organizational commitment to the project. The TROPIC initiative provided a win-win situation, with participants expanding skills in EIPM and policy development, organizations increasing EIPM capacity, and researchers providing data to inform policy.

## Background

Overweight and obesity is a major public health issue both globally [1,2] and among Pacific populations [3-6]. Many Pacific Island countries have experienced a rapid nutrition transition from subsistence crops to imported foods, and this effect has been compounded by the sedentary behaviors associated with urban lifestyles [7,8]. The prevalence of adult overweight/obesity (BMI > 25 kg is over 75% in Nauru, Samoa, American Samoa, Cook Islands, Tonga and French Polynesia) [9,10]. Researchers and policymakers around the world are struggling in their search to find effective strategies to combat the growing prevalence of obesity [11-14]. The need for policy interventions that can potentially improve environments and support healthy diets and physical activity levels has been widely recognized [15], but few evidence-informed policy initiatives have been implemented [16-19]. Evidence can inform policies during several phases of development: the inception of the policy, review of policy options [20], and impact assessment of potential risks and benefits of a policy [21].

More policies need to be implemented in order to improve food and physical activity environments, especially policies developed by non-health sectors [22]. The need for policy measures to prevent obesity was emphasized at the 2011 UN High-Level Summit on Non-Communicable Diseases (NCDs) [23], as well as in the Moscow declaration [5]. Although much has been done in the Pacific region for NCD prevention and control, there is scope for a more coordinated approach to developing policies that build a healthier environment and promote healthier choices. It is important that these policies are informed by the best evidence available [24,25]. For example, since the Pacific Ministers of Health recommended that countries implement legal and fiscal measures which can promote healthy diet and physical activity to combat chronic disease [26], very little action has occurred. Although the current government has a promising strategic framework to streamline policymaking processes in Fiji, there is no emphasis on the use of research evidence in policymaking in any of the key government documents which refer to policy [27]. While there is a great need to entrench a culture of using sound evidence in decision/policymaking, there was no formal evaluation of the use of research findings in policymaking in Fiji at baseline.

While the importance of using research findings in decisionmaking at the policy level has been increasingly recognized [28-30], the process remains a challenge [31,32], with considerable gaps between researchers and policymakers in terms of implementing effective strategies that increase the translation of research evidence into effective policy and planning. One way to increase the use of evidence in policy development is to employ a knowledge brokering approach to bridge the gap between researchers and potential evidence users such as policy makers [5] and advocates [30,33]. Knowledge brokering has been defined as an individual, team or organization who promotes interaction between researchers and end-users [34] or a ‘linkage agent’ [35]. Van Kammen and colleagues see this interactive process as producers and users of knowledge co-producing feasible and research-informed policy options [36]. There is a wide spectrum of knowledge brokering roles, including: ensuring a mutual understanding of goals and cultures, collaborating with end-users to identify problems, developing capacity for and facilitating the use of evidence-informed policymaking [34], sourcing, interpreting and adapting evidence [34,37], facilitating access to evidence, commissioning evidence synthesis [36], and monitoring the impact of evidence-informed policymaking (EIPM) [36,38]. Given the broad range of skills required of a knowledge broker, it was elected to have a knowledge brokering team with a range of complementary skills. A team versus individual approach provides not only complementary skills but also a broader perspective [39]. A multidisciplinary perspective is advantageous when developing evidence-informed policies in a range of departments. Dobbins *et al*., in a randomized controlled trial to examine three knowledge translation activities to promote the use of evidence-informed decisionmaking (EIDM) concluded that the most important factors were relationship development, ongoing support, tailored approaches, and providing opportunities for developing capacity at individual and organizational levels [38]. While increased levels of interaction between researchers and policymakers can facilitate the use of research findings [40-44], very few studies have examined the processes involved in knowledge brokering [45]. The Translational Research on Obesity Prevention in Communities (TROPIC) project was a natural extension of the Pacific Obesity Prevention in Communities (OPIC) project that generated substantial data on adolescent obesity through the delivery of multi-faceted interventions in school and community settings in Fiji [3], Tonga [6], New Zealand [46], and Australia [47], as well as examining sociocultural, socioeconomic and policy factors. The research question for the subsequent TROPIC project was: Can a knowledge brokering approach advance evidence-informed policy development to improve eating and physical activity environments in Fiji. This paper examines the knowledge brokering processes employed in the TROPIC project, using process evaluation. Process evaluation is defined for the purpose of this paper as a process to monitor and document program implementation and can aid in understanding the relationship between specific program elements and program outcomes [48].

## Methods

The TROPIC project was a three-year study, funded by the Australian Agency for International Development (AusAID) on an Australian Development Research Awards grant. The project was approved by the Deakin University Human Research Ethics Committee, the Fiji Health Research Committee, and the Fiji National Research Ethics Review Committee. TROPIC was managed by the Fiji School of Medicine in Suva (now part of Fiji National University), under the Pacific Research Centre for the Prevention of Obesity and Non-communicable Diseases (C-POND) and Deakin University. The knowledge brokering team comprised: a knowledge broker who coordinated the recruiting, interventions and follow-up activities in Fiji; a part-time research fellow who assisted in the intervention and evaluation phases; a consultant based at Deakin University (Australia) who worked on site for part of the project and provided remote support when off-site; and an advisory group (comprised of four individuals with prior experience in one or more participating organization). The terms ‘knowledge brokering team’ and ‘TROPIC team’ will be used interchangeably.

**Figure 1:**
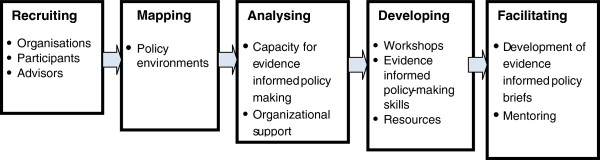
Different phases of TROPIC project.

The knowledge brokering team worked collaboratively with four government departments and two non-government organizations. Participants within each organization worked together on TROPIC activities, as well as having some opportunities to work across participating organizations toward the end of the project. Knowledge brokering activities comprised five phases: recruiting organizations and individuals; mapping policy environments; analyzing organizational capacity and support for EIPM; developing EIPM skills; and facilitating development of evidence-informed policy briefs (Figure 1).

### Recruiting

#### Organizations

The first phase of the project was to select the organizations using purposive sampling. Selection criteria that were based on other study designs [49,50], and consultation with local experts and the TROPIC team included: the potential of an organization to make or positively influence policies that could improve food and/or physical activity environments; representation of different demographic groups to ensure potential policy reach (*e*.*g*., across key ethnic and religious groups; urban and rural settings); capacity to release staff for TROPIC activities; potential to apply and share policymaking knowledge and skills within the organization; and potential to build capacity in order to develop a critical mass of policy-makers who utilize EIPM [51]. The last two criteria were based on documentation within corporate plans of formal and informal capacity building programs for their own personnel and for the community.

In order to gain the understanding and support of both government ministers and directors of non-government organizations (NGOs), concept papers detailing the benefits of TROPIC partnerships were tailored for each individual organization [52]. Ten government and non-government organizations were identified as potential participants. We aimed to recruit six organizations. Eight organizations were prioritized to be approached first based on the previously indicated criteria. We over-selected potential organizations because we expected some invitees to decline the invitation. High-level meetings subsequently took place, conducted with government ministers, permanent secretaries (lead civil servants) in government organizations, or chief executive officers from NGOs. Two of the six government organizations approached declined the invitation to participate in the study due to lack of organizational resources (time, staff). Both of the NGOs that were approached agreed to participate.

#### Focal points

Each participating organization nominated a senior staff member to act as a focal point (contact person). Focal points had two key roles within his/her organization: identification and recruitment of participants with either policymaking or advocacy roles; and co-ordination of the TROPIC program.

#### Individual participants

Between five and twelve staff members in each organization who were engaged in policy development were nominated as participants by focal points.

#### Advisors

Four advisors who were familiar with the policymaking process in one or more of the participating organizations were recruited by the TROPIC team on the basis of their experience and expertise in policymaking processes. Their roles were to advise the TROPIC team on policy processes and organizational culture throughout the knowledge brokering process [52].

#### Experts

Experts who had in-depth knowledge and recent experience with one or more participating organizations provided an ‘outsider’s perspective of the organizational culture, as well as identifying resources and providing support for EIPM in each of the participating organizations.

### Mapping policy environments

At the start of the TROPIC project, key national and organizational documents were examined in order to identify the priorities of each organization and potential policy topics that could enhance the health of the population by reducing obesity and/or obesogenic environments. Documents included the Government’s Charter for Change [27,53], as well as each organization’s strategic plan and corporate plan. For example, a need for policies to create an enabling environment for physical activity was identified in the Fiji NCD Strategic plan [54].

Each potential partner organization was ‘mapped’ to determine: mission statements; existing programs and policies in place to address obesity prevention (*e*.*g*., relating to nutrition and/or physical activity); policy gaps; organizational resources; and factors that could potentially influence decisionmaking (*e*.*g*., power relations; budgets). Topics for policy briefs were then negotiated individually with each organization to ensure that they aligned with both organizational plans and the specific goals of TROPIC [52].

### Analyzing capacity

Semi-structured interviews were conducted with individual participants (n = 49) to identify their knowledge about and experience with evidence, research, evidence-informed decisionmaking and/or policy development [55,56]. Semi-structured interviews were also conducted with five experts in each participating organization to obtain their perceptions of whether and how TROPIC has impacted their organization. A questionnaire [57] was also administered to participants prior to EIPM activities in order to assess baseline evidence-informed decisionmaking skills and perceived support for EIPM [21]. See Mavoa *et al*., 2012, for details of the questionnaire [52].

### Developing evidence-informed policymaking skills

The next stage of TROPIC was to work with participants to build their skills in EIPM [21,55]. In line with other studies, TROPIC aimed to: increase the capacity of participants to acquire/access, assess, adapt and apply evidence [55]; use research-based evidence to inform selected policy topics [31,58]; support participants to develop a policy brief [20]; support participants to present a policy brief to higher level senior officers [20,59] and to seek the each organization’s endorsement and implementation of the policy; and where possible, develop a second evidence-based policy brief with reduced support from the TROPIC team. This capacity building approach included four to six workshops conducted separately for each of the six organizations at a time and place suitable to them. Each workshop ranged from two to three and a half hours’ duration. Workshops were delivered at two- to four-week intervals, depending on each group’s availability. The workshops comprised presentations and discussions about: what constitutes evidence; what constitutes a policy and the policy cycle in Fiji; selecting policy topics that aligned with organizational and national goals; and acquiring/accessing, assessing, adapting and applying evidence.

To increase participants’ access to relevant evidence, information was provided on relevant online databases, as well as local sources of evidence. Additionally, the TROPIC team facilitated access to the World Health Organization Hinari program [60] that provides health-related literature. Participants who were enrolled in local universities were encouraged to use their existing student access for library resources and management systems (*e*.*g*., Endnote). General support for acquiring/accessing, assessing and adapting evidence was also provided to each participant through face-to-face meetings, as well as by telephone and electronic communications; this one-to-one support builds on learnings from the workshop sessions.

### Facilitating development of evidence-informed policy briefs

In line with Lavis and colleagues [61], one of the main targeted outcomes for TROPIC was the development of evidence-informed policy briefs. Policy topics were aligned to both national and organizational strategies, and addressed obesity either directly or indirectly by targeting changes in food or physical activity environments [52]. Specific policy topics were negotiated between participants, their individual heads of department, and the TROPIC team. Templates outlining the processes for developing policy briefs (Table 1) were developed to guide participants; while templates for each organization had some commonalities, other components were tailored to organizational requirements. At least two members of the TROPIC team reviewed multiple drafts of each policy brief and provided iterative reviewer feedback to each participant that was coordinated by the knowledge broker. The TROPIC team provided less intensive support during the formulation of the second policy brief. Once the policy briefs reached a sufficiently high standard, participants gave oral presentations and then submitted the written brief to high-level officers/decision-makers in their organization. Participants were provided with templates for their oral presentations and given an opportunity to practice their upcoming presentations with guidance from the TROPIC team.

**Table 1:** Headings for the policy brief template

**Nos**.	**Subtitle**
**1**	Policy topic
**2**	Executive summary
**3**	Objectives
**4**	Background
**5**	Definitions
**6**	Key evidence findings
**7**	Relevant legislations and authorities
**8**	Plans for implementations
**9**	Proposed cost
**10**	Health impact assessment
**11**	Monitoring and evaluation
**12**	Recommendations
**13**	References

### Process data

Process diaries were kept by TROPIC team members. All intervention-related activities such as workshops, meetings, etc., were recorded by individual knowledge brokering teams at the end of each day, using a data collection proforma. In line with Waters *et al*. [62], the diaries detailed all interactions and activities that occurred with participants, focal points and other personnel in participating organizations. Entries included: planning and implementation (description), processes (how the activity was conducted), dose (scale and duration of the activity), reach (how many people were involved in the activity) and frequency (how often an activity was delivered). All intervention data were recorded on an excel database, and contact times were analyzed accordingly.

## Results

The results are discussed under the knowledge brokering activities used in Table 2.

**Table 2:** Summaries of knowledge brokering activities

**Category**	**Description of activities**	**Outcomes and comments**
**Recruiting partner organizations**		
Organizations	Followed selection criteria, prepared concept papers that advocate for obesity and NCD and policy environment High level officers appointed Focal Points to coordinate programs with researchers	Twelve government and non-government organizations identified, eight approached, six were endorsed and recruited while two declined.
Participants	49 senior officers recruited across organizations	Emails, phone calls and face to face meetings held
Advisors	Followed selection criteria of those familiar with the policy-making process	Four advisors were recruited
**Mapping policy environment**		
Source of information	Searched in government websites: organizational mission, vision statements, corporate and strategic plans, annual reports, received hard copies from contacts within organizations	Access to Roadmap for democracy and sustainable socio-economic development (SSED), Peoples Charter for Change, MDGs, Corporate plans, strategic plans.
**Analyzing organizational capacity and support for evidence**-**informed policy**-**making (EIPM)**		
Semi-structured interviews	Conducted with individual participants and experts in each participating organization	49 participants and 5 experts consented and interviewed
Questionnaires	Developed, piloted and administered to participants prior to evidence-informed policy-making activities	49 participants returned completed questionnaire
**Developing evidence**-**informed policy**-**making skills**		
PowerPoint Presentations	Developed Master presentations for evidence-informed policy-making	PowerPoint presentations tailored to each organization needs prepared
Fact Sheets	Developed guide on how to acquire evidence using different search engines, access and analyze	50 Fact sheets distributed to participants
Template for policy briefs	Developed template for writing policy briefs	5/6 organizations used the template, one organization had its own template prior to engagement with TROPIC
Template for presentation of policy briefs	Developed template for PowerPoint presentation of policy briefs	2/6 organizations made oral presentations using template provided, 2 others discussed policy briefs in a closed meeting, the 2 NGOs discussed proposed policy briefs via phone and send through email.
Evidence-informed policy-making	Conducted 27 workshops across 6 organizations, 45% of participants attended at least 1 workshop and 55% attended whole workshop series	2 organizations selected a second more senior participant group because of high staff turnover and recognition of the importance of having senior officers develop their EIPM skills
Skill-based workshop (small group)	Conducted 26 workshops with small group of participants on skill training, 35% of participants attended	These included those who either missed out a session or need skill training in referencing, use of Endnote software, professional writing and health impact assessment.
**Facilitating development of evidence-informed policy briefs**		
One-to-one meeting	156 meetings conducted with 40 participants across 6 organizations, assisted in acquiring evidence and writing briefs	77% of participants attended; 262 hours of support provided from individual TROPIC team
Small Group meeting	30 meetings conducted across 6 organizations, generally 2 participants, encouraging continuity of EIPM against tight work schedule, and completing policy briefs.	35 hours of support provided
Policy brief meeting	98 meetings conducted across 6 organizations, assisted individuals or small groups in reviewing policy briefs, in writing and presentation skills to high level officers.	52% of participants attended; 141 hours of support provided
Telephone-base counseling	85 phone calls made to participants across organizations in shaping the development of policy briefs	21 hours of support provided
Email, SMS	799 emails sent to participants supporting the development of policy briefs, counseling and encouraging continuity of EIPM, 33 txt messages reminding participants on approaching deadline for next draft of briefs	290 hours of support provided from individual TROPIC team
Selection of policy topics	Facilitated selection of 35 policy topics by participants	35% of participants completed at least one policy brief; 20 policy briefs completed and presented to high level officers
Sourced materials: Technical	Facilitated access to Hinari website, Endnote software and Use of IT Laboratory from Ministry of Health for practical learning of acquiring evidence using different search engines	Sourced Hinari from Ministry of Health to its participants, referred student access to those enrolled in other local institutions in Fiji

### Recruiting partner organizations

A total of 49 participants were recruited, of whom 41% were senior managers, 45% middle managers, and 14% junior managers (Table 3). A total of 65% of participants were aged between 31 to 50 years, and 51% were female. A total of 55% of participants (n = 27) attended all workshops and developed one or two policy briefs over the 12 to 18 month intervention; 63% of participants who completed the entire program produced one or more policy briefs. The remaining 45% of participants attended at least one workshop, citing heavy workloads, taking up scholarships for further study, resignation from their post, or relocation either within Fiji or overseas as reasons for failing to complete the project.

**Table 3:** Demographic profile of participants

**Names**	**Org 1 n = 13**	**Org 2 n = 12**	**Org 3 n = 5**	**Org 4 n = 10**	**Org 5 n = 5**	**Org 6 n = 4**	**Total n = 49**
**Ethnic Group**							
iTaukei (Indigenous Fijians)	10	8	5	8	3	3	37 (76 %)
Fijians (of Indian descent)	3	3		2	2	1	11 (22 %)
Others		1					1 (2%)
**Gender**							
Male	6	11	3	1	3	0	24 (49%)
Female	7	1	2	9	2	4	25 (51%)
**Reasons for non-completion**							
Resignation or relocation either within Fiji or overseas	5	4		3	1		13 (27%)
Work pressure or study		5	1			3	9 (18%)
**Management level**							
Junior	1	1	-	-	2	3	7 (14%)
Middle	4	10	2	5	1	-	22 (45%)
Senior	8	1	3	5	2	1	20 (41%)

The TROPIC project provided ongoing professional development for each organization, especially for junior officers. Two organizations selected a second wave of more senior officers into the program, on advice from the knowledge brokering team once it had become evident that the more junior participants would have few opportunities to apply their newly acquired EIPM skills (Table 2). Importantly, the addition of more senior participants increased the likelihood that skills developed during TROPIC could be utilized and sustained beyond the project.

### Mapping policy environments and analyzing capacity

Many participants were aware of key government documents that were used in planning but had limited access and minimal use in reporting, monitoring and evaluating existing programs.

However almost all participants were able to explicitly discuss the existing problems that concerned them, and to suggest policy options but could not identify the research to support these.

Some never understood the importance and use of research evidence in decision/policymaking, and very few used the literature. A few participants collected data as part of their job description, and some of those undertaking post-graduate studies had specific research projects.

Some participants referred to the data routinely collected as evidence and knew little about the difference between statistics and published data, or the difference between evidence and policy. This information was used to inform the workshop contents.

### Developing evidence-informed policymaking skills

A total of 27 workshops were conducted between February 2010 and March 2011, with several weeks between each workshop. Given the competing work priorities for most senior officers (*e*.*g*., attending high level meetings, workshops and supervising field staff), last minute re-scheduling of some workshops was often required; this affected project timelines and costs. Every opportunity was taken by the TROPIC team to support participants who missed a particular workshop session or who needed further training in EIPM skills, for example accessing unfamiliar databases or using Endnote.

A standard template for constructing policy briefs was developed for five organizations (Table 1). However, one organization elected to use a template that was already in place. This template limited the development of EIPM skills because it did not accommodate the presentation of evidence-based arguments to support the proposed policy topic. A template was also developed for participants to use when preparing presentations to higher-level officers.

### Facilitating development of evidence-informed policy briefs

A total of 20 policy briefs were submitted to a higher-level management within each organization. A wide range of policy topics relating to food and physical activity environment were chosen by participants, including ones targeting food production, food pricing, settings and marketing. They also targeted physical activity environments. The topics were selected by participants based primarily on their interest area and expertise, and overall were intended to reduce the cost of, and increase the accessibility to healthier products, and to increase cost of and reduce accessibility to less healthy products, along with policies to improve the environment for physical activity. Where possible, advocacy briefs were prepared by NGOs in tandem with policy briefs and submitted to government organizations prior to the presentation of policy briefs on the same topic.

**Figure 2:**
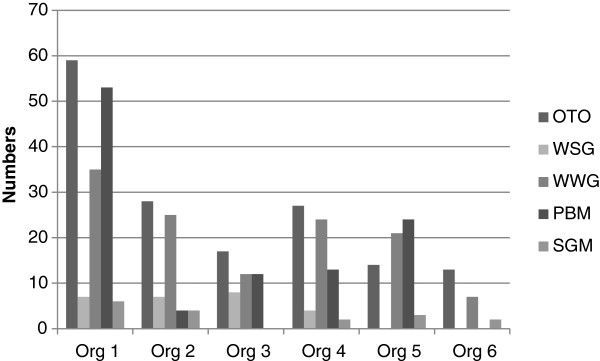
Frequency and types of engagement.

An average of 30 hours per participant was expended by TROPIC team members in workshop preparation and delivery, mentoring and supporting policy brief development during the 12 to 18 month intervention period (Table 2). These included attending workshops, one-on-one sessions where individuals were assisted to access/acquire evidence and develop policy briefs, small group activities to support professional writing and presentation skills, electronic feedback and telephone interactions. The majority of participants preferred one-on-one visits and were assisted in acquiring data (accessing different types of search engines), accessing the best literature to inform their briefs, actual writing of policy briefs, use of endnotes and other skills. Figure 2 shows how activities differed across participating organizations.

All participants had access to email, which served as the primary mode of communication among the TROPIC team in terms of general reminders about upcoming activities or deadlines, as well as the provision of feedback on policy briefs. Mobile and landline phone conversations were also common modes of communication; the number of phone calls made was not fully documented.

While the internet is an increasingly important and popular source of evidence, some organizations had no or limited access to online databases. Given the limited resources, access to and experience with different electronic databases and through various search engines (*e.g*., Pubmed, Medline) promoted considerable interest among participants.

## Discussion

The TROPIC knowledge exchange program delivered a large number of activities and initiatives over the 12- to18-month intervention period. TROPIC presented a unique opportunity for participants to acquire/access, assess, adapt and apply evidence-based information and to transfer this into policy briefs in a low to middle income Pacific nation. The TROPIC engagement resulted in the presentation of 20 policy briefs to higher-level decision makers within each organization. Policy briefs included data on the prevalence of obesity among the Pacific populations, obesogenic environment and integrating EIPM [63] using a knowledge brokering approach [45,55]. These are similar to themes highlighted by others who have examined the roles of knowledge-brokers [31,38].

The processes of engaging researchers and knowledge-users in the TROPIC program is quite different from others [34,64].The involvement of higher-level officers such as government ministers or permanent secretaries (deputy ministers) in government departments and executive officers and/or directors in non-government organizations at the initial stage of the project (consultations and endorsement of partnership) was a critical factor in gaining access to key people (departmental heads or focal points). These key personnel were selected by higher-level officers in each of the agencies who were most intimately involved in the development of policies or training.

In each organization, the early engagement of key personnel facilitated recruitment, ensured that TROPIC timelines were mutually acceptable, and built policy-developer-researcher relationships that were critical for both active engagements in the TROPIC program and the embedding of EIPM beyond the project. The involvement of the designated focal points greatly contributed to the smooth coordination of participants, as well as organization of workshops and meetings (venues, times).

Individual participants’ level of engagement during TROPIC varied. Work pressures were cited as one of the main reasons for the larger than expected proportion of participants who did not complete all intervention activities, similar to that found elsewhere [65-67]. While all selected organizations remained supportive throughout the course of the project, only one participant had her workload reduced in order to actively engage in TROPIC activities. Natural disasters (a cyclone; two major floods; typhoid epidemic) and unexpected diversions compromised the completion of policy briefs. Participation may have been higher if the workshops had been delivered away from the workplace and/or were conducted in a block.

The organization culture of some participating organizations may not have been optimal to support EIDM. Two organizations appeared ready to support EIDM, and this was reflected in the relatively high number of policy briefs that were completed. The detail of completed policy briefs is the subject of a separate paper. The building of EIPM skills was intended to move beyond theoretical understanding to the development of practical skills/competencies in developing evidence-informed policy briefs and submitting them to higher levels. Further building of EIDM capacity and the development of structures and processes that support EIPM is expected to enhance the sustainability of this critical approach to policymaking as supported by others [49,51,56]. The specific TROPIC initiatives that aimed to develop and embed a culture of EIPM will be the subject of a separate paper.

The TROPIC team provided a high intervention dose to support the completion of policy briefs. This high dose required could be explained by participants: overestimating their EIPM skills when entering the intervention; prioritizing other work priorities over EIDM workshops and/or policy brief development; and giving low priority to policy brief development, especially given that production of policy briefs was not a core output for most participants.

The inclusion of policy brief development in individual job descriptions through corporate plans and ongoing capacity building through in-house training are promising strategies to embed EIPM. Policy brief completion was evidence of skill development, TROPIC team support and, importantly, successful communication processes that built trust and maintained good relationships between the knowledge brokering team and participating organizations [41,45]. The building of good relationships between researchers and end-users is an essential criterion for success [63].The interaction between the knowledge brokering team, individual participants and high-level senior officers within and across organizations was an important part of the knowledge brokering processes. The TROPIC project was unique in its use of a number of complementary approaches that strengthened the knowledge brokering role. These included tailoring of concept papers, use of advisors, and use of a standard template for policy briefs. However, the high staff turn-over in all six organizations limited the continuity of workshops and the completion of policy briefs. The need for flexible time-tabling of activities was also important as organizations had multiple competing priorities. The knowledge brokering team also underestimated the time required to negotiate policy brief topics, intensifying the problems of trying to sustain a high level of participation throughout the intervention process. Unexpectedly, there was little overall awareness of relationships between obesity, the environment, and the economic impact of non-communicable diseases. The team accommodated this by providing relevant information for participants in the form of concept papers or supporting literature.

## Conclusion

The TROPIC project was able to successfully recruit and retain organizations and their staff for this innovative research initiative. Strong commitments to involvement were shown by many of the participants, as reflected by their attendance at workshops, involvement with mentoring and completion of policy briefs. The combination of support styles (workshops, mentoring and draft review) was well-used by most participants, and this type of flexible, tailored and intense delivery system may be of value elsewhere, especially in low to middle income countries with limited resources. The TROPIC initiative provided a win-win situation, with participants expanding skills in EIPM and policy development, organizations increasing EIPM capacity, and researchers providing data to inform policy. The team involved has secured funding to continue some of the capacity building elements of TROPIC, and is also developing plans to address sustainability of the approach.

## Competing interests

The authors declare that they have no competing interests.

## Authors’ contributions

GW was the knowledge broker, project coordinator, and lead writer. HM reviewed the literature, led the design process, and commented on the manuscript. WS provided valuable advice on TROPIC activities, as well as revising the manuscript.PK contributed to the study design and critically reviewed the manuscript. MM, JS and MMc reviewed the manuscript, and BS was the senior researcher overseeing all aspects of the project. All authors read and approved the final manuscript.
